# Self-efficacy, social support and oral health-related quality of life in patients with kidney transplantation and under hemodialysis

**DOI:** 10.1186/s12882-024-03889-0

**Published:** 2024-12-02

**Authors:** Bero Luke Vincent Ernst, Deborah Kreher, Daniel Patschan, Rainer Haak, Thomas Ebert, Jonathan de Fallois, Gerhard Schmalz

**Affiliations:** 1 Department of Cariology, Endodontology and Periodontology, University of Leipzig, Leipzig, Germany; 2 intHERCon GmbH, Goettingen, Germany; 3 Department of Cardiology, Angiology and Nephrology, Klinikum Brandenburg, Medizinische Hochschule Brandenburg, Brandenburg, Germany; 4 Department of Nephrology, Rheumatology and Endocrinology, University of Leipzig, Leipzig, Germany; 5 Department of Conservative Dentistry and Periodontology, Brandenburg Medical School (MHB) Theodor Fontane, Geschwister-Scholl-Straße 36, Brandenburg/Havel, D 14776 Germany

**Keywords:** Oral health, Oral health-related quality of life, Kidney replacement therapy, Hemodialysis, Kidney transplantation, Self-efficacy

## Abstract

**Background:**

Aim of this questionnaire-based cross-sectional study was to compare self-efficacy, social support, oral hygiene-related self-efficacy (OHRSE) and oral health-related quality of life (OHRQoL) between patients under chronic hemodialysis (HD) and patients after kidney transplantation (KTx) as well as a healthy comparison group (HC).

**Methods:**

Patients under HD were recruited during their routine outpatient dialysis therapy, KTx patients during their maintenance appointment and HC patients during their regular dental check-up in the dental clinic. General self-efficacy, the OHRSE, social support (F-SozU-K14) and the OHRQoL (OHIP-G5) were assessed by specific validated questionnaires. The survey was performed by one experienced dentist.

**Results:**

44 HD, 40 KTx and 45 HC patients were included, between which the age and gender distribution was comparable (*p* > 0.05).

With a median of 1.5 [IQR: 0–3], HD patients showed higher OHIP-G5 than the participants in KTx group (*p* < 0.01). Similarly, a significant difference was found between KTx (0, [0–0.5]) and HC (0, [0–3]; *p* < 0.01).

HD patients showed a lower sum score of OHRSE, tooth-brushing, interdental-cleaning and dental-visit self-efficacy than the HC (*p* < 0.01). Similarly, HD patients had a lower sum score of OHRSE, tooth-brushing and dental-visit self-efficacy than the KTx group (*p* < 0.01). Moreover, the KTx group had a lower interdental-cleaning self-efficacy (*p* < 0.01) and sum score (*p* = 0.02) than the HC. The sum score of the general self-efficacy was comparable between the three groups (*p* = 0.19). The F-SozU-K14 revealed higher values in KTx (65.40 ± 5.33) compared with HD (60.95 ± 9.28) and HC group (61.71 ± 9.24; *p* = 0.03).

Only in the KTx group, a significant association between F-SozU-K14 and OHIP-G5 was revealed (*p* = 0.05).

**Conclusions:**

Patients under HD show a reduced OHRSE compared to KTx and HC and a slightly reduced OHRQoL compared to KTx patients. While general self-efficacy was comparable between groups, social support of HD patients was lower than of KTx patients. The OHRSE and OHRQoL might receive increased attention in dental care of HD patients.

## Background

The oral health situation of patients under kidney replacement therapy, particularly under chronic hemodialysis (HD), is a topic of high practical relevance; recent literature summarizes, that patients with end-stage kidney disease, especially with kidney replacement therapy, suffer from a high prevalence of carious tooth decay [1], as well as more severe periodontal diseases [2]. This problem is not restricted to the oral cavity. A recent meta-analysis concluded that especially periodontitis would be significantly associated with all-cause mortality in patients with chronic kidney diseases [3]. In this context, a cross-sectional study showed that periodontitis would be associated with malnutrition, inflammation and atherosclerosis in patients with end-stage kidney disease [4]. Thus, it is not surprising that improvements in oral health care of those patients appear mandatory.

Possibly the most relevant issue in context of oral health is the patient itself. It is well known from decades that the patient´s oral hygiene behavior is crucial for the therapy and prevention of oral diseases, especially periodontitis [5]. In this context, it has repeatedly been shown that oral health behavior is reduced in patients under kidney replacement therapy, especially under HD, what might be one main cause of their worse oral health situation [6–8]. Thereby, the patient perspective appears of relevance. During dialysis vintage time, patients under HD experience a worsening in oral health and, at the same time, a decrease in their attention on oral health related topics [9]. The oral health-related quality of life (OHRQoL) seems often unaffected in those patients, although the oral disease burden is high [10]. This leads to the hypothesis that those patients undergo an oral health response shift during their kidney replacement therapy, similar as for patients with other chronic general diseases [11]. Taken together, patient-specific factors like oral health behavior alongside with oral health perception, appear highly relevant in context of the high prevalence of oral diseases in those patients. Only one previous study addressed this issue more in depth; a cross-sectional study by Fallahi et al. (2023) showed that the perceived self-efficacy and perceived barriers were relevant issues in context of dental cleaning behavior of HD patients [8]. Beside of this, that field of research appear understudied, yet.

Accordingly, this current study aimed in a questionnaire-based evaluation of self-efficacy, social support, oral hygiene-related self-efficacy and OHRQoL of patients under kidney replacement therapy. Thereby, the main purpose was to assess, whether patients under HD would show worse results in the upper mentioned parameters compared with individuals after kidney transplantation (KTx) and a healthy comparison group.

## Methods

### Study design

This current study was designed as a monocentric cohort study, which has been reviewed and approved by the ethics committee of the medical faculty of Leipzig University (306/22-ek). All participants were informed about the study and gave their written informed consent. The recruitment of study participants was conducted between January 1st 2023 and October 31st 2023. With regard to the study aims, which were stated in the background section, the following hypotheses were formulated: I) patients under HD show worse self-efficacy, social support, oral hygiene-related self-efficacy and OHRQoL compared to the two other groups. II) Associations between OHRQoL and the other parameters are apparent.

### Participants

Participants out of three groups were recruited during the current study. Therefore, in- and exclusion criteria were formulated.

#### Participant in- and exclusion criteria

In general, the following criteria were defined for the participants in this current study:

- age of 18 years or older
- ability to provide informed consent

Additionally, the following exclusion criteria were formulated as well:

- inability to undergo the survey due to worse health status
- severe dementia (based on medical history)
- history of organ transplantation, except for kidney
- insufficient German language skills, which did not allow an appropriate answer of the respective questions

Furthermore, group-specific criteria were considered, as shown below.

#### HD group

For the HD group, patients under chronic hemodialysis with a chronic kidney disease of stage 5D (CKD 5D) were asked for their voluntary participation during their dialysis appointment at the Department of Nephrology, Leipzig University. Specific inclusion criterion was a chronic HD in the upper mentioned center. The participants were recruited subsequently.

#### KTx group

In a second group, patients after kidney transplantation (KTx) were recruited during their regular outpatient appointment within their maintenance in the Department of Nephrology, Leipzig University. Specific inclusion criterion was a KTx (at least 6 months ago). Patients with KTx, who recently underwent a HD therapy were excluded from the study.

#### HC group

For comparison, a systemically healthy group (HC) was recruited. Those individuals were patients attending the Department of Cariology, Endodontology and Periodontology, Leipzig University, for their routine dental check-up appointment. The absence of systemic diseases, especially any form of kidney diseases, was confirmed by checking the medical history of the patients.

To increase comparability of the different cohorts, the age and gender distribution of the comparison group was matched to the HD group as good as possible.

#### Sample size

The sample size was determined as follows: the primary parameter was the OHRQoL (OHIP-G5 sum score), whereby a difference of at least one point in median should be evaluated with a power of at least 0.8 and an alpha of 0.05. This resulted in a required sample of at least 32 individuals per group. For the current study, it was aimed to include at least 40 individuals per group to support statistical robustness of the sample.

### Questionnaires

In this current survey, four questionnaires were used. The patients were surveyed by one experienced dentist. Only completed questionnaires were considered for statistical analysis.

#### OHRQoL

For assessment and interpretation of the OHRQoL, this current study followed the recommendations for use and scoring of oral health impact profile versions [12] as well as for the standardization of dental patient-reported outcomes measurement [13]. The OHRQoL was assessed by the German short form of the oral health impact profile (OHIP-G5) [14]. With the OHIP G-5, five different perceived impacts related with teeth, mouth or dentures were evaluated. Thereby, the following answer options exist: very often = “4”, fairly often = “3”, occasionally = “2”, hardly ever = “1”, and never = “0”. Thus, a lower value indicates a higher OHRQoL of the participant. Beside of the sum score of OHIP G5, the four dimensions “oral function” (question 1 and 2), “orofacial pain” (question 3), “orofacial appearance” (question 4) and “psychosocial impact” (question 5) were considered.

#### Oral hygiene-related self-efficacy

Lastly, a specific oral hygiene-related self-efficacy (OHRSE) questionnaire was applied, as previously introduced by Wölber et al. (2015) [15], which based on the Dental self-efficacy scales [16]. Thereby, nineteen questions were rated on a four-point scale as follows: “completely confident not to” (1 point), “fairly confident not to” (2 points), “fairly confident to” (3 points), “completely confident to” (4 points). The questions are separated into tooth-brushing self-efficacy (6 questions), inter-dental cleaning self-efficacy (6 questions) as well as dental visit self-efficacy (7 questions).

#### General self-efficacy

To assess the general self-efficacy, the questionnaire by Schwarzer and Jerusalem was applied [17]. This questionnaire consists of ten different questions, which are answered by a four point scale between 1 (don´t agree) and 4 (fully agree). Therefore, the higher the value, the higher the self-efficacy.

#### Social support

For the evaluation of social support, the short form of the social support questionnaire (F-SozU-K14) was used [18]. This questionnaire consists of fourteen questions, which reflect different issues related with social support. All of those questions can be answered on a five point scale between 1 (don´t agree) and 5 (fully agree). Accordingly, a higher value indicates a higher social support.

### Statistical analysis

All analyses were conducted by using SPSS for Windows, version 24.0 (SPSS Inc., US). Firstly, Kolmogorov–Smirnov-Test showed that none of the tested variables were normally distributed, why non-parametric tests were used exclusively. Comparing two independent samples, Mann–Whitney-U-test was applied. More than two independent samples were tested by Kruskal–Wallis test. Categorical or nominal data were compared by chi-square test, respectively. To reveal associations between OHIP-G5 and the other tested variables, an ANOVA was applied. The significance level was set at *p* < 0.05.

## Results

### Participants

Out of 100 HD patients, which were treated in the study center, 44 individuals with an age of 63.23 ± 14.78 years and 56.8% male gender were included. Forty patients after KTx, out of 80 individuals, who were asked for participation (mean age: 60.23 ± 14.45 years, 57.5% male) were included. The healthy comparison group consisted of 45 participants with a mean age of 63.49 ± 11.52 years and 55.6% males. The age and gender distribution were comparable between groups (*p* > 0.05). In the KTx group, a lower amount of smokers was found, compared with the two other groups (*p* < 0.01, Table 1).

**Table 1:** Patient characteristics, presented as mean value ± standard deviation, mean value (range) or percentage

**Parameter **		**HD (*n*=44)**	**KTx(*n*=40)**	**HC (*n*=45)**	***P*-value**
**Gender (male) [n (%)]**		25 (56.8)	23 (57.5)	25 (55.6)	0.98
**Age in years (mv ± SD)**		63.23 ± 14.78	60.23 ± 14.45	63.49 ± 11.52	0.81
**Smoking habits [n (%)] **	Smoker > 10 cig./day	7 (15.9)	0 (0)	9 (20)	**<0.01**
	Smoker ≤ 10 cig./day	11 (25)	4 (10)	13 (28.9)	
	non-smoker	26 (59.1)	36 (90)	23 (51.1)	
**General disease, conditions and medication[n (%)] or (mv ± SD)**	Diabetes mellitus	19 (43.2)	11 (27.5)		0.13
	hypertonia	27 (61.4)	34 (85)		0.03
	pAVK	3 (6.8)	4 (10)	-	0.70
	stroke	5 (11.4)	5 (12.5)		0.99
	Number of medications	6.61 ± 3.93	10.5 ± 6.08		<0.01
	Body-mass-index (BMI)	27.05 ± 6.96	25.6 ± 3.98		0.53
**Time under hemodialysis / since transplantation in years **(mv ± SD)		5.4 ± 4.8	7.3 ± 4.7		-

*HD* hemodialysis, *KTx* kidney transplantation, *HC* healthy comparison

### Oral health-related quality of life

**Fig. 1:**
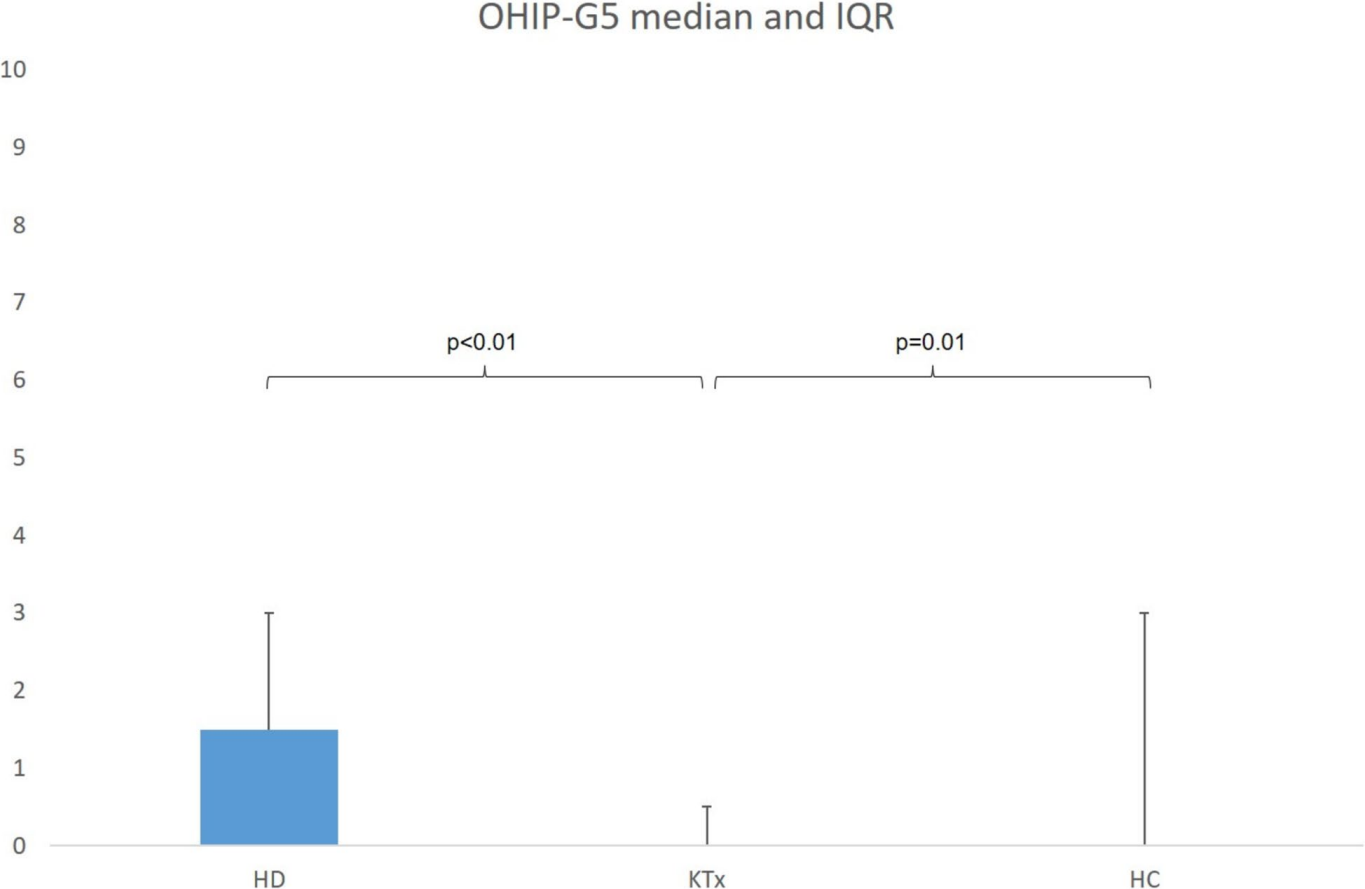
Sum scores of OHIP-G5 questionnaire between groups. The figure shows Median as blue bar chart and inter quartile range (IQR) as lines for the respective groups. The *p*-values represent the significances in group comparisons

With a median of 1.5 [IQR: 0–3], HD patients showed higher OHIP-G5 than the participants in KTx group (*p* < 0.01). Similarly, a significant difference was found between KTx (0, [0–0.5]) and HC (0, [0–3]; *p* < 0.01), as shown in Fig. 1. This significant difference was also confirmed for the questions regarding difficulty chewing any foods (*p* < 0.01) and felt less flavor in food (*p* = 0.02, Table 2). Thus, the dimension oral function was particularly less affected in KTx group compared to the other two groups (HD and HC). Additionally, the dimension orofacial appearance, represented by question 4, was significantly more affected in HD and HC compared to KTx (Table 2).

**Table 2:** Results of the OHIP-G5 reflecting the oral health-related quality of life. Results are given as mean value ± standard deviation [median, 25th and 75th percentile]

Question	HD (*n * = 44)	KTx (*n* = 40)	HC (*n* = 45)	*P*-value
**Difficulty chewing any foods (indicator of Oral Function)**	0.68 ± 1.09 [0, 0, 1]	0 ± 0 [0, 0–0]	0.44 ± 0.87 [0, 0, 1]	** < 0.01**
**Felt less flavor in food (indicator of Oral Function)**	0.25 ± 0.61 [0, 0–0]	0 ± 0 [0, 0–0]	0.24 ± 0.65 [0, 0–0]	**0.02**
**Painful aching in your mouth (indicator of Orofacial Pain)**	0.30 ± 0.79 [0, 0–0]	0.30 ± 0.79 [0, 0–0]	0.47 ± 0.97 [0, 0–0]	0.47
**Felt uncomfortable about the appearance (indicator of Orofacial Appearance)**	0.59 ± 1.00 [0, 0, 1]	0.23 ± 0.70 [0, 0–0]	0.69 ± 1.10 [0, 0, 1]	**0.03**
**Difficulty doing your usual jobs (indicator of Psychosocial impact)**	0.11 ± 0.44 [0, 0–0]	0 ± 0 [0, 0–0]	0.11 ± 0.32 [0, 0–0]	0.11

*HD* hemodialysis, *KTx* kidney transplantation, *HC* healthy comparison

### Oral hygiene-related self-efficacy (OHRSE)

**Fig. 2:**
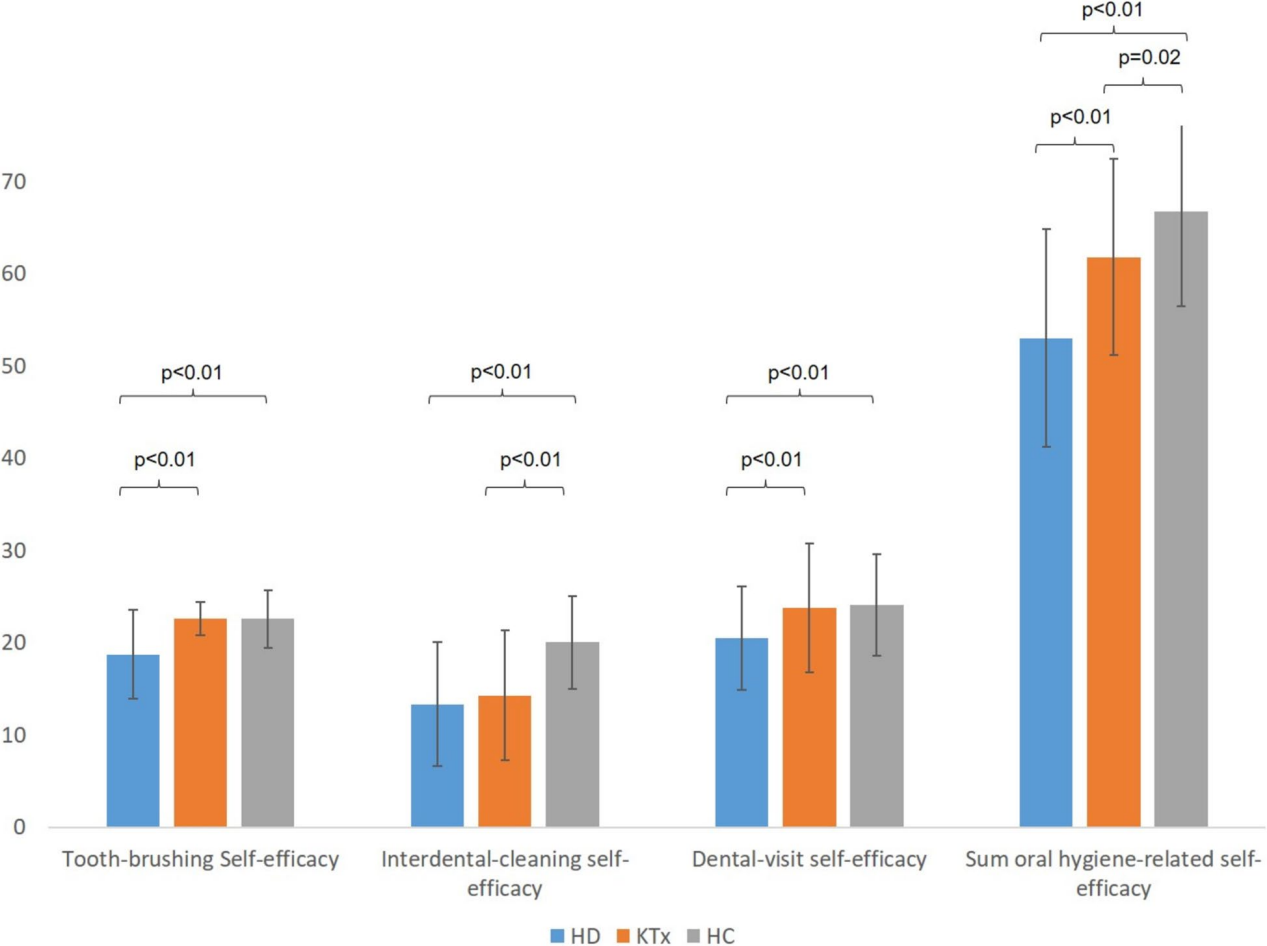
Sum scores of the oral hygiene-related self-efficacy between the three groups. The bar charts show the mean values, while the lines represent the standard deviation

As depicted in Fig. 2, the OHRSE differed between groups, what was found for the sum score, the three sub-scores, as well as all of the questions (Fig. 2, Table 3). Thereby, HD patients showed a lower sum score, tooth-brushing, interdental-cleaning and dental-visit self-efficacy than the HC (*p* < 0.01). Similarly, HD patients had a lower sum score, tooth-brushing and dental-visit self-efficacy than the KTx group (*p* < 0.01). Moreover, the KTx group had a lower interdental-cleaning self-efficacy (*p* < 0.01) and sum score (*p* = 0.02) as the HC (Fig. 2).

**Table 3:** Results for oral hygiene-related self-efficacy questionnaire. Results are given as mean value ± standard deviation

Question	HD (*n* = 44)	KTx (*n* = 40)	HC (*n* = 45)	*P*-value
***How confident are you, that you brush your teeth in the following situations?***				
**When you are tired in the evening**	2.86 ± 1.03	3.55 ± 0.78	3.71 ± 0.66	** < 0.01**
**When you are not going to a dentist in near future**	3.64 ± 0.78	3.98 ± 0.16	3.82 ± 0.65	**0.01**
**When you are on holiday**	3.45 ± 0.90	3.95 ± 0.22	3.89 ± 0.49	** < 0.01**
**When you have a lot of work**	3.27 ± 1.04	3.93 ± 0.27	3.89 ± 0.27	** < 0.01**
**When you have a headache**	3.20 ± 1.02	3.83 ± 0.50	3.58 ± 0.89	** < 0.01**
**When you feel ill**	2.77 ± 0.94	3.40 ± 0.63	3.69 ± 0.73	** < 0.01**
***How confident are you, that you clean your proximal surfaces in the following situations?***				
**When you are tired in the evening**	2.09 ± 1.18	2.25 ± 1.13	3.22 ± 0.97	** < 0.01**
**When you are not going to a dentist in near future**	2.39 ± 1.30	2.48 ± 1.22	3.53 ± 0.79	** < 0.01**
**When you are on holiday**	2.41 ± 1.32	2.40 ± 1.28	3.49 ± 0.82	** < 0.01**
**When you have a lot of work**	2.30 ± 1.30	2.45 ± 1.24	3.49 ± 0.84	** < 0.01**
**When you have a headache**	2.20 ± 1.23	2.43 ± 1.24	3.20 ± 1.08	** < 0.01**
**When you feel ill**	1.98 ± 1.11	2.28 ± 1.18	3.11 ± 1.07	** < 0.01**
***How confident are you, that you visit the dentist as often as advised?***				
**When a dentist does not invite you to visit regularly**	2.98 ± 1.11	3.53 ± 0.91	3.67 ± 0.77	** < 0.01**
**When you have no dental symptoms**	2.93 ± 1.25	3.52 ± 0.91	3.60 ± 0.78	**0.01**
**When you have money problems**	3.23 ± 1.05	3.78 ± 0.70	3.38 ± 1.09	**0.01**
**When you are busy**	2.93 ± 1.07	3.57 ± 0.87	3.49 ± 0.94	** < 0.01**
**When you are unable to make an appointment with a known dentist**	3.77 ± 1.43	4.02 ± 1.42	3.80 ± 1.27	** < 0.01**
**When you have earlier unpleasant experiences**	2.45 ± 1.15	3.20 ± 1.09	3.24 ± 1.03	** < 0.01**
**When you are frightened of painful interventions**	3.11 ± 0.99	3.70 ± 0.79	3.51 ± 0.92	** < 0.01**

*HD* hemodialysis, *KTx* kidney transplantation, *HC* healthy comparison

### General self-efficacy and social support

**Fig. 3:**
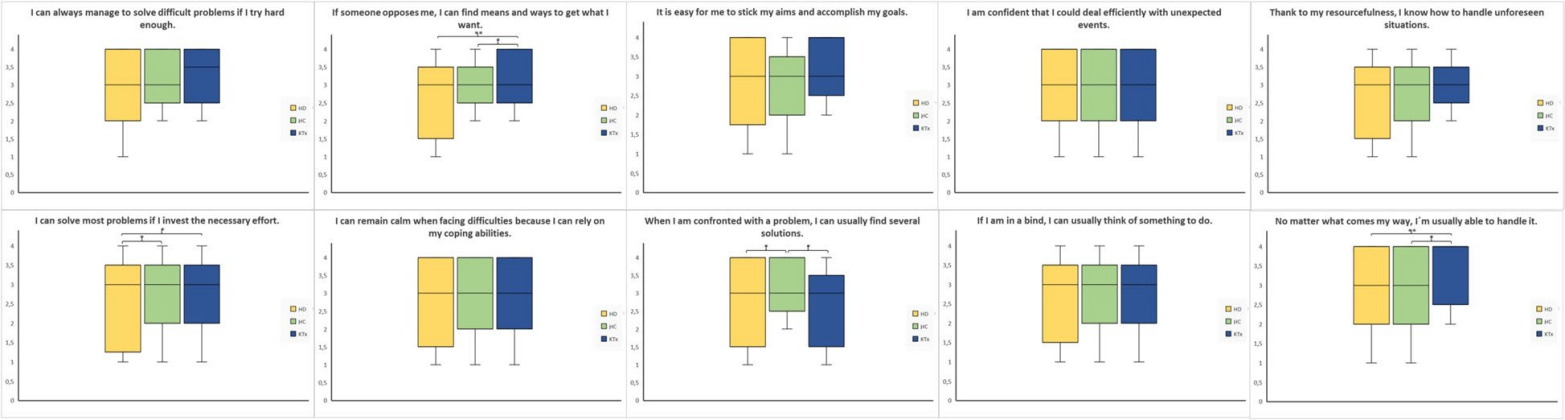
Results of the general self-efficacy questionnaire. Results are given as box-plots for the three groups HD (hemodialysis), HC (healthy comparison) and KTX (kidney transplantation). In case of significant differences between the groups, those are presented as * for *p* < 0.05 and ** for *p* < 0.01

The sum score of the general self-efficacy was comparable between the three groups with mean values for HD of 29.18 ± 6.41, KTx of 31.65 ± 3.86 and HC of 31.04 ± 5.36 in sum score (*p* = 0.19). As Fig. 3 shows, several questions differed between the groups. Thereby, four questions showed lower values in the HD group, i.e. “If someone opposes me, I can find means and ways to get what I want.” (*p* < 0.01), “I can solve most problems if I invest the necessary effort.” (*p* = 0.03), “When I am confronted with a problem, I can usually find several solutions.” (*p* = 0.03) and “No matter what comes my way, I´m usually able to handle it.” (*p* < 0.01; Fig. 3).

**Fig. 4:**
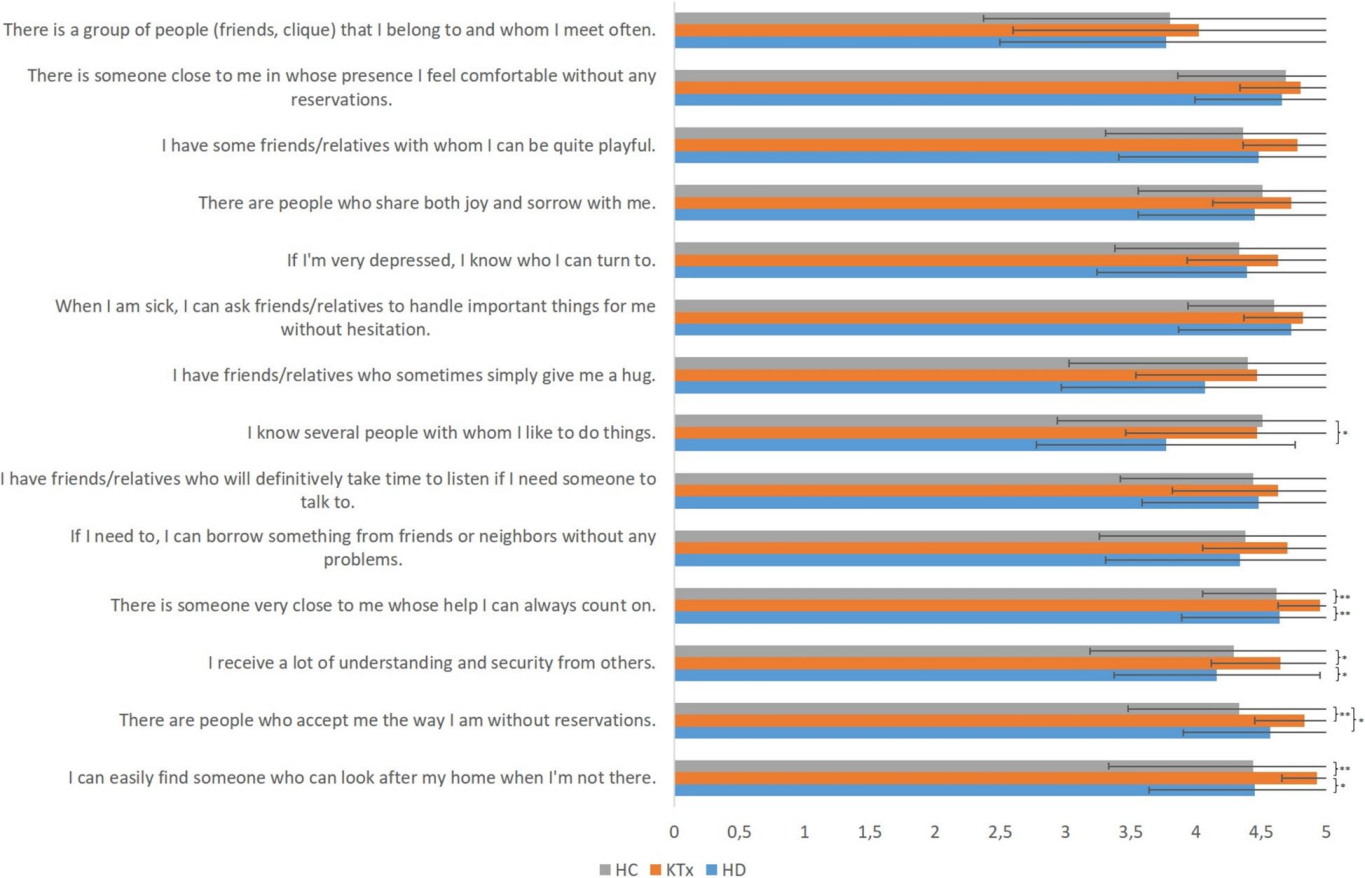
Findings of the F-SozU-K14 representing the social support. The values are shown as means (bar chart) and standard deviations for patients under hemodialysis (HD), after kidney transplantation (KTx) and healthy comparison group (HC). Significant differences are shown as * for *p* < 0.05 and as ** for *p* < 0.01

Regarding social support, the F-SozU-K14 revealed higher values in KTx (65.40 ± 5.33) compared with HD (60.95 ± 9.28) and HC group (61.71 ± 9.24; *p* = 0.03). This difference was also found for some of the fourteen questions, as shown in Fig. 4.

### Relationship between oral health-related quality of life and the other parameters

Table 4 shows the *p*-values of the examination of potential associations between OHIP-G5 and the other survey results. Thereby, only in the KTx group, a significant association between F-SozU-K14 and OHIP-G5 was revealed (*p* = 0.05). Other associations could not be confirmed.

**Table 4:** *P*-values for potential associations between OHIP-G5 values and self-efficacy, social support und oral hygiene-related self-efficacy within the three groups

Parameter	HD (*n* = 44)	KTx (*n* = 40)	HC (*n* = 45)
**General self-efficacy**	0.13	0.20	0.79
**F-SozU-K14**	0.24	**0.05**	0.85
**Tooth-brushing self-efficacy**	0.42	0.53	0.09
**Interdental-cleaning self-efficacy**	0.39	0.22	0.11
Dental-visit self-efficacy	0.77	0.68	0.44
Sum oral hygiene-related self-efficacy	0.43	0.38	0.96

*HD* Hemodialysis, *KTx* kidney transplantation, *HC* healthy comparison

## Discussion

The main results of this current study showed a worse OHRQoL of patients under HD compared with patients under KTx. Moreover, the OHRSE was worse in HD patients compared to the two other groups. Social support was highest in KTx patients, whereby this parameter was associated with OHRQoL.

Against the background of a response shift in oral health topics, which was presumed for patients under HD, this study based on the hypotheses that self-efficacy, OHRQoL and OHRSE would be reduced in HD patients. This hypothesis was only partly confirmed. Patients under HD have a high burden due to their general disease and therapy, resulting in frailty, fatigue, depression, anxiety and a reduction in quality of life [19–21]. Thereby, the self-efficacy of patients under HD is often reported to be reduced and has already been identified as a relevant target in health care [22, 23]. In the basic definition, self-efficacy is defined as “one's belief in the ability to perform the desired functions” [24]. Due to the health impairments, a reduction in self-efficacy of HD patients is plausible. However, although some questions showed significant differences, the general self-efficacy did not significantly differ between HD, KTx and HC in the current study. This finding was unexpected, whereby a clear explanation is missing.

In contrast, the OHRSE was clearly decreased in HD patients, affecting all of its dimensions, questions and the sum score (see Fig. 2). It has been reported that a lower OHRSE would be related with less successful daily plaque control [25]. In a prospective cohort study, Wölber et al. (2015) showed that OHRSE is an influential factor on oral hygiene behavior as well as predictor of oral hygiene [15]. While comparable literature for patients with kidney diseases are missing, as this is the first study in this particular field, the reduced OHRSE appears to be one potential explanation of the insufficient oral health behavior as well as high prevalence of oral diseases in this cohort, which has been described previously [1, 2, 6–8]. However, this assumption is limited by the fact that this current study was only a survey, without a clinical oral examination of the included patients. Another interesting finding is the difference between HD and KTx patients in tooth-brushing and dental visit self-efficacy, as well as in the sum score of OHRSE. It is known that KTx leads to an improvement of quality of life [26] and a remarkable systemic health benefit [27]. Furthermore, patients prior to organ transplantation (including KTx and other solid organs) are regularly referred to a dentist to eliminate potential oral foci [28]. This might lead to a higher attention on oral health, more regular dental visits and tooth-brushing and might also affect the OHRSE. Considering the finding that HD patients show a reduced OHRSE, this issue might be addressed in oral health care as one important element to improve their oral situation.

The main focus of this manuscript was the OHRQoL. Thereby, HD patients showed a worse OHRQoL than KTx patients, which might have a similar explanation as the difference in OHRSE. Interestingly, the OHRQoL of HD patients did not differ significantly from the HC group. It has already been described previously, that patients under HD often show nearly unaffected OHRQoL in German cohorts, although their oral conditions were insufficient [9, 29]. This has already been explained by a potential response shift, whereby the burden of dialysis therapy might cover the potential burden of oral diseases. Considering reference values for OHIP-G5 in German general population, as provided by John et al. 2004, only the value of the HD group in this current study was slightly higher than the reference [30]. This argues for a slightly reduced OHRQoL in the HD group, and a non-reduced OHRQoL in the two other groups. Surprisingly, neither the general self-efficacy, nor the OHRSE were associated with the OHRQoL in this study. Thus, the perception of OHRSE appears not to affect the oral health perception of patients under kidney replacement therapy, what might be a further supportive argument for their response shift. Considering the dimensions of OHIP-G5 in the current study, it was conspicuous that only the dimensions oral function and orofacial appearance differed significantly between groups. The missing differences between HD and HC seems a further argument for the response shift in HD, what would not explain the less affected dimensions in KTx patients. Within a systematic review, the OHRQoL of patients under kidney replacement therapy has already been discussed with regard to the different dimensions [10]. This systematic review assumed primarily an affection of the psychosocial impact dimension in patients under kidney replacement therapy, which would be potentially associated with the general and oral disease burden of the patients [10]. This reads contradictory with the current study´s results, where the psychosocial impact did not differ between groups. Especially for patients after KTx, no affection of oral function and psychosocial impact was confirmed.

This current study considered one more parameter, i.e. the social support of the participants. Thereby, the F-SozU-K14 revealed that patients after KTx perceived a higher level of social support compared with the two other groups. It has been reported that accessing a kidney transplant required patient engagement, motivation and also stable social support [31]. Especially in context of psychological issues, a functional social network and support appear important before and after KTx [32]. On the other hand, social support is sometimes impaired in patients under HD and thereby related with self-management and sense of coherence [33]. It appears conceivable, that especially patients with a good and stable social support meet the challenges before, during and after transplantation. This might result in a higher perception of social support in this post-transplant cohort. The level of social support was associated with the OHRQoL in the KTx group. It is presumed, but still unclear, whether social support has a potential buffering effect in context of OHRQoL [34]. However, it could be one reason for the better OHIP-G5 scores in KTx compared with the other groups. This should be further elaborated in subsequent studies. Overall, social support could be potentially promising approach to improve the oral health care concepts of patients under HD, especially under consideration of OHRSE and OHRQoL.

### Strengths and limitations

This questionnaire-based study is the first one, which assessed self-efficacy, social support, OHRSE and OHRQoL of patients under kidney replacement therapy. The inclusion of a reasonable cohort of patients and a HC with comparable age and gender distribution is a further strength. The chosen questionnaires were validated instruments and an experienced dentist performed all of the surveys, ensuring a correct understanding of all questions. The major limitation is the absence of a clinical oral examination. Without clinical data on oral hygiene and oral disease, those parameters cannot be included in the interpretation of the results. The cohort was only monocentric and biased by the motivation of patients to participate, what is always a mandatory perquisite for study participation, but might positively influence the results of self-efficacy, OHRSE and OHRQoL by excluding severely ill und unwilling individuals. The difference in smoking behavior and medication between HD and KTx are further limiting factors. Moreover, the study is limited by its cross-sectional character. To draw meaningful conclusions, a prospective design is needed. The comparison group consisted of patients attending the dental clinic for regular dental check-up, making this cohort not representative for general population.

Taken together, the current study´s findings can serve as an interesting basis for future research in the field, but the investigation still has a pilot character and should therefore be interpreted like this.

## Conclusions

Within the limitations of the current study, HD patients show a reduced OHRSE compared to KTx and HC and a slightly reduced OHRQoL compared to KTx patients. The general self-efficacy was comparable between groups. Furthermore, social support of HD patients was lower than of KTx patients. The OHRSE and OHRQoL might receive increased attention in dental care of HD patients to elaborate more patient-centered interventions in the future.

## Data Availability

The datasets used and/or analyzed during the current study are available from the corre-sponding author on reasonable request. The data are not publically available, because of the psedonymisation and data protection guidelines according to the ethics approval.

## Abbreviations

CKD5D: Chronic kidney disease stage 5D requiring kidney replacement therapy
HD: Hemodialysis
KTx: Kidney transplantation
OHIP: Oral health impact profile
OHRQoL: Oral health-related quality of life
OHRSE: Oral hygiene-related self-efficacy
